# A robust walking detection algorithm using a single foot-worn inertial sensor: validation in real-life settings

**DOI:** 10.1007/s11517-023-02826-x

**Published:** 2023-04-18

**Authors:** Gaëlle Prigent, Kamiar Aminian, Andrea Cereatti, Francesca Salis, Tecla Bonci, Kirsty Scott, Claudia Mazzà, Lisa Alcock, Silvia Del Din, Eran Gazit, Clint Hansen, Anisoara Paraschiv-Ionescu, for the Mobilise-D consortium

**Affiliations:** 1 Laboratory of Movement Analysis and Measurement (LMAM), École Polytechnique Fédérale de Lausanne, Lausanne, Switzerland; 2 Department of Electronics and Telecommunications, Politecnico Di Torino, Turin, Italy; 3 Department of Biomedical Sciences, University of Sassari, Sassari, Italy; 4 Interuniversity Centre of Bioengineering of the Human Neuromusculoskeletal System, Sassari, Italy; 5 Department of Mechanical Engineering and Insigneo Institute for in Silico Medicine, University of Sheffield, Sheffield, UK; 6 Translational and Clinical Research Institute, Faculty of Medical Sciences, Newcastle University, Newcastle Upon Tyne, UK; 7 Center for the Study of Movement, Cognition and Mobility, Neurological Institute, Tel Aviv Sourasky Medical Center, Tel Aviv, Israel; 8 Department of Neurology, University Medical Center Schleswig-Holstein Campus Kiel, Kiel, Germany

**Keywords:** Walking detection, Real-world, Foot-worn sensor, Continuous wavelet transform, Adaptive threshold

## Abstract

Walking activity and gait parameters are considered among the most relevant mobility-related parameters. Currently, gait assessments have been mainly analyzed in laboratory or hospital settings, which only partially reflect usual performance (i.e., real world behavior). In this study, we aim to validate a robust walking detection algorithm using a single foot-worn inertial measurement unit (IMU) in real-life settings. We used a challenging dataset including 18 individuals performing free-living activities. A multi-sensor wearable system including pressure insoles, multiple IMUs, and infrared distance sensors (INDIP) was used as reference. Accurate walking detection was obtained, with sensitivity and specificity of 98 and 91% respectively. As robust walking detection is needed for ambulatory monitoring to complete the processing pipeline from raw recorded data to walking/mobility outcomes, a validated algorithm would pave the way for assessing patient performance and gait quality in real-world conditions.

**Graphical Abstract:**

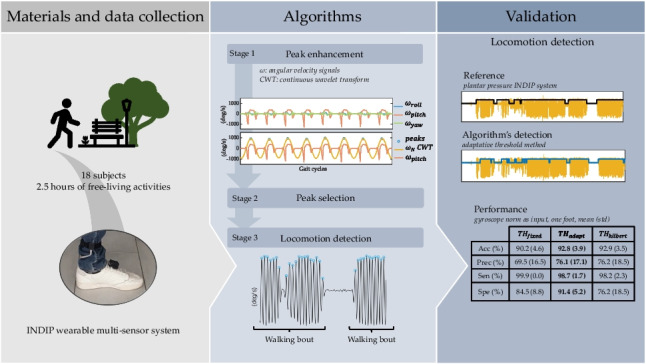

**Supplementary Information:**

The online version contains supplementary material available at 10.1007/s11517-023-02826-x.

## Introduction

The use of wearable sensors offers a valuable opportunity to obtain objective and valid mobility data in free-living conditions [1, 2]. Walking activity and gait parameters are considered among the most relevant mobility-related parameters [3, 4]. Gait speed has been referred to as the sixth vital sign [5] and has been shown to be a reliable marker of functional decline and mobility [6, 7]. The assessment of walking in real-life conditions and the effects of specific interventions or medications on patient mobility are hence of great interest [8]. In this context, automatic detection of walking bouts (WBs) (also referred as to gait /walking periods) before further gait analysis and speed estimation is necessary.

The most commonly used algorithms for ambulatory locomotion detection are based on trunk-mounted (chest/lower back) inertial measurement units (IMUs) and use different approaches such as threshold-based methods [9–12], zero-crossing methods [13, 14], and pattern recognition [15]. One of the main advantages of using a sensor on the chest/lower back is the possibility to detect other physical activities besides locomotion, such as lying, sitting, standing, and postural transitions [16–18]. The advantage of lower back position is the estimation of step length, cadence and gait speed [19]. A wrist-mounted sensor is also commonly used to assess daily activity [20–22] because it is practical to use and convenient for participants. Although recent algorithms allow estimation of gait speed based on lower back or wrist position [23, 24], a foot/shank-mounted IMU may provide detailed spatio-temporal parameters with clinically acceptable accuracy [25, 26]. This is because the assumption of zero-velocity-update can be exploited to reduce errors affecting the estimation of spatial parameters [25, 27–29].

In a systematic review, Vienne et al. [2] identified seven clinical criteria for semiological descriptions of gait quality in patients with neurological disorders, i.e., springiness, sturdiness, smoothness, steadiness, stability, symmetry, and synchronization [2]. With the exception of stability and synchronization, requiring an IMU attached to the trunk, all the other gait criteria can be computed from an IMU attached to the shank or foot. Therefore, in the context of clinical studies, in which accurate locomotion identification and detailed gait analysis are critical for outcome evaluation, the foot can be considered as an appropriate sensor location.

Currently, gait assessments have been mainly analyzed in laboratory or hospital settings [2]. In a systematic review, Vienne et al., 2017 reported that of the 78 included studies, only 9 examined gait when patients were in their natural home environment, and only one measured gait with a foot-worn sensor. Recently, discrepancies have been demonstrated in mobility parameters measured in unsupervised and in laboratory settings [30]. Short walking tests performed in the laboratory provide a snapshot of patients’ capacity, which does not reflect their usual performance (i.e., real world behavior). There is growing consensus that unsupervised assessments for long-term mobility evaluation in real-life conditions can complement supervised walking tests, and contribute to individualized clinical decisions [30, 31]. The main challenge in unsupervised measurements is the need for an accurate gold standard reference system for algorithm validation. Video recordings are often used as a reference for labelling real-life activities [32]. However, manual labelling is very time-consuming, cumbersome, and should be performed independently by multiple observers to allow for cross-validation. Recently, a multi-sensor wearable system, which integrates pressure insoles with multiple IMUs and infrared distance sensors (INDIP), have been developed to be used as references for real-world experimentation and locomotion detection [33, 34]. Instrumented insoles, combined with IMUs, provide reliable identification of initial and final foot contacts, which is essential for walking bout detection and gait analysis [33].

Given the above-mentioned considerations, a robust detection of walking bouts for ambulatory monitoring based on foot-mounted IMUs is necessary to complete the processing pipeline from raw recorded data to walking/gait-related mobility outcomes. A validated algorithm would pave the way for evaluating patient performance and gait quality in a natural home environment. Therefore, using the INDIP pressure sensor system as a reference, the aim of this study is to validate a walking bout detection algorithm using data recorded in real-life like environments with a foot-mounted IMU. The algorithm was evaluated for various sensor configurations, i.e., for one IMU (one foot) or two IMUs (two feet), and for 3D acceleration or 3D angular velocity signals.

## Methods

### Materials and data collection

**Fig. 1:**
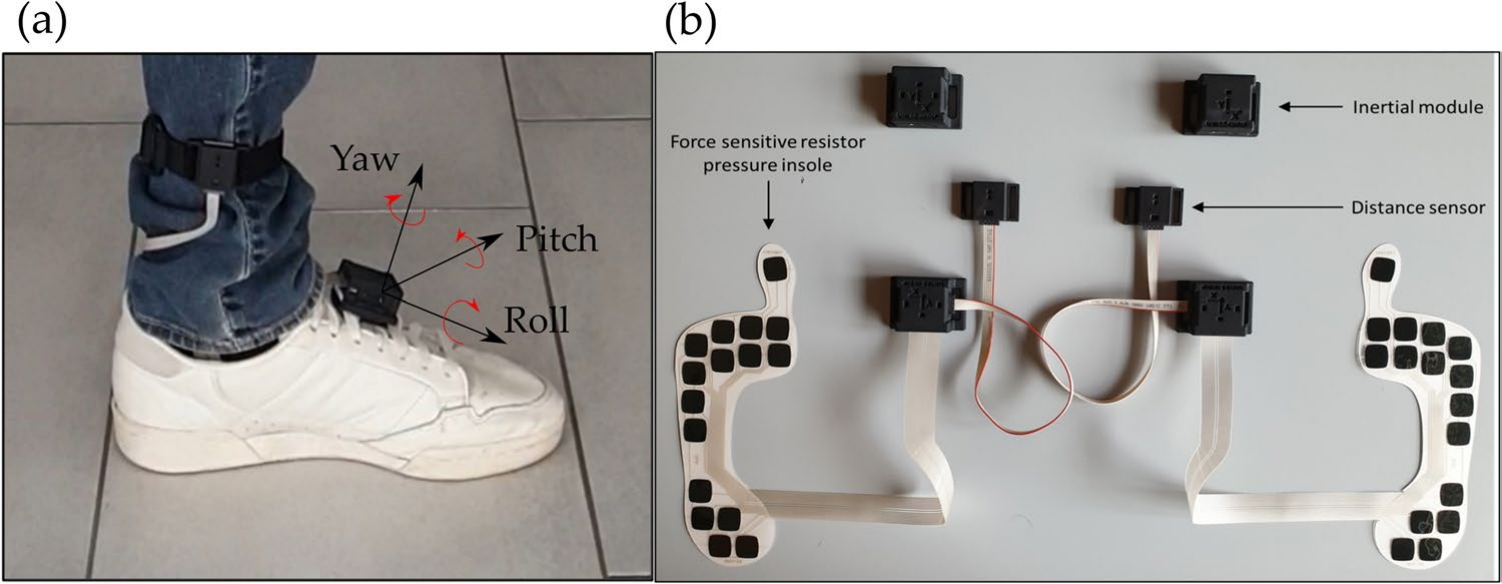
Multi-sensor wearable system (INDIP); (**a**) INDIP system attached on the shoe, and (**b**) picture of two INDIP sensors (right and left feet), which integrate force sensitive resistor pressure insoles, inertial modules (IMUs), and infrared distance sensors

Data used to evaluate the performance of the proposed WBs detection algorithm were recorded in 18 healthy subjects (10 healthy young (HY) and 8 healthy adults (HA)) who performed 2.5 h of free-living activities. The study was approved by the University of Sheffield Research Ethics Committee (application number 029143). During the 2.5 h of measurement, subjects were asked to: (1) rise from a chair and walk to another room; (2) walk to the kitchen and make a drink; (3) walk up and down a set of stairs; (4) walk outside, if possible for a minimum of 2 min; and (5) if walking outside, walk up and down an inclined path. Each participant was equipped with a INDIP wearable multi-sensor system (Fig. 1). The INDIP system includes on each foot an IMU (3D accelerometer, ± 16 g; 3D gyroscope, ± 2000 dps; 3D magnetometer), 2 distance sensors, and two 16-elements plantar pressure insoles. In this study, acceleration and angular velocity, recorded at 100 Hz from shoe-mounted IMUs, were used to develop a new algorithm for step/stride and WBs detection. The performance of this algorithm was validated using the INDIP system, and dedicated algorithms, as a reference (ground truth) [33].

### Algorithm implementation

Step or stride detection from foot-worn sensors are generally based on the detection of peaks associated to consecutive mid-swing, heel-strike [28] or toe-off [35] instants in the pitch angular velocity obtained from the gyroscope signal. However, those detections are highly sensitive to sensor placement and orientation, and require a calibration procedure [25]. Thus, in this study, the 3D angular velocity norm, (*ωN,* Eq. 1) is used to overcome the calibration step:

1 $$\omega N= \sqrt{{\omega }_{roll}^{2}+ {\omega }_{pitch}^{2}+ {\omega }_{yaw }^{2}}$$

where ${\omega }_{roll}$, ${\omega }_{pitch}$ and ${\omega }_{yaw}$ are the components of the angular velocity signal recorded by the gyroscope around the 3D rotation axes. In addition to the gyroscope signal, we also tested the performance of the algorithm when using the acceleration norm (*aN*) as input, making our methodology usable for 3D accelerometer only.

**Fig. 2:**
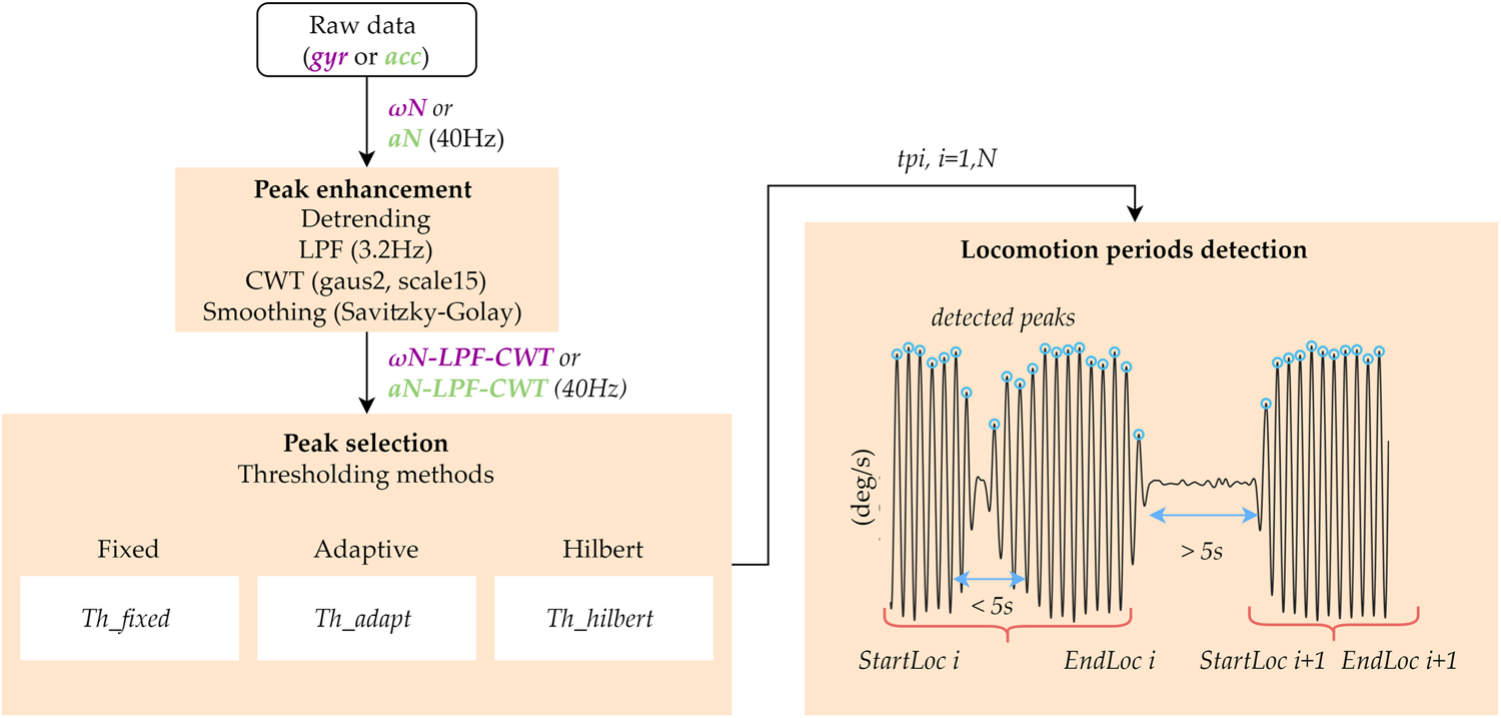
Flowchart of the walking bouts detection algorithm for one-foot IMU. *ωN*: raw angular velocity norm; *aN*: raw acceleration norm; LPF: low-pass filter; CWT: continuous wavelet transform; $${TH}_{fixed}$$: *fixed* threshold ($${TH}_{fixed}=100 deg/s$$ or $${TH}_{fixed}=0.5 g$$); $${TH}_{adapt}$$: *adaptive* threshold based on the percentile of the obtained amplitude distribution of peaks detected above the fixed threshold ($${TH}_{fixed})$$; $${TH}_{hilbert}$$: *Hilbert* threshold based on the *Hilbert* envelope and percentile of the obtained amplitude distribution of peaks detected; $${tp}_{i}$$: time of peak occurrence; *StartLoc i*: start of the walking bout *i*; *EndLoc i*: end of the walking bout *i*

When using one IMU on one foot, only stride-related information from the instrumented foot can be extracted. The acceleration and angular velocity norm demonstrate periodic patterns with peaks generated by the contact with ground (i.e., heel-strike and toe-off) and the mid-swing events at each stride. To enhance these periodic patterns and reduce the effect of movement artefacts, a *peak enhancement* stage was applied to obtain a signal containing stride-related information. Then, a *peak selection* stage using threshold-based approaches was designed to select the peaks corresponding to the strides [36]. A similar approach was used when considering two IMUs (one on each foot) to obtain step-related information. Finally, a *walking bout detection* method was applied using the selected strides or steps (Fig. 2). In the following paragraphs, we described these three stages with *ωN* used as the input signal.

#### Peak enhancement

**Fig. 3:**
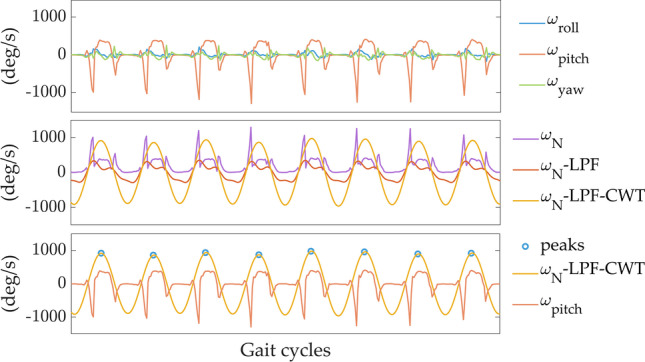
Angular velocity signals recorded with the 3D gyroscope on one foot during several gait cycles for one subject. The top panel shows the raw angular velocity signals around the three axes (roll, pitch, yaw). The middle panel shows the raw angular velocity norm (*ωN*, magenta), the signal after detrending and LPF (*ωN-LPF*, red), and the signal after continuous wavelet transform (*ωN- LPF-CWT*, yellow). The bottom panel shows the pitch angular velocity, the continuous wavelet transform (*ωN- LPF-CWT*, yellow), and the detected strides. Strides are identified as maxima corresponding to mid- swing events (blue circle) with an amplitude higher than a certain threshold (${TH}_{fixed}$, ${TH}_{adapt}$, or ${TH}_{hilbert}$)

The objective of this first stage is to obtain a signal that contains improved stride-related information. This peak enhancement method should be robust to various gait patterns/impairments. The raw angular velocity norm (*ωN*) is first down-sampled to 40 Hz to decrease the processing time when data is recorded in long-term monitoring protocols. Then, the signal is detrended and low-pass filtered (FIR filter, *n* = 120 coefficients, cutoff frequency: *Fc* ≈3.2 Hz) to obtain *ωN-LPF* [10]. The cutoff frequency *Fc* was chosen to smooth the signal by removing the high frequency noise. As FIR filters have a linear phase shift, the shape of the waveform is not modified, however, a delay is introduced. To eliminate the delay and obtain zero-phase distortion, the filter is applied to *ωN* twice using the Matlab function *Filtfilt.* Subsequently, the continuous wavelet transform (cwt, scale 15, gauss2 wavelet in Matlab) is used as a smoothing and differentiation procedure [37], allowing a stride-related peaks enhancement consistent among various impaired or atypical gait patterns. The cwt time-scale was set to 15 to be adapted to stride detection. Finally, additional slight smoothing is performed using a linear Savitzky-Golay filter. The processed signal is referred to as *ωN-LPF-CWT*. An illustrative example of the peak enhancement procedure for the signals recorded from one-foot IMU is shown in Fig. 3, and in Supplementary material A when two IMUs are used (one on each foot).

#### Peak selection

**Fig. 4:**
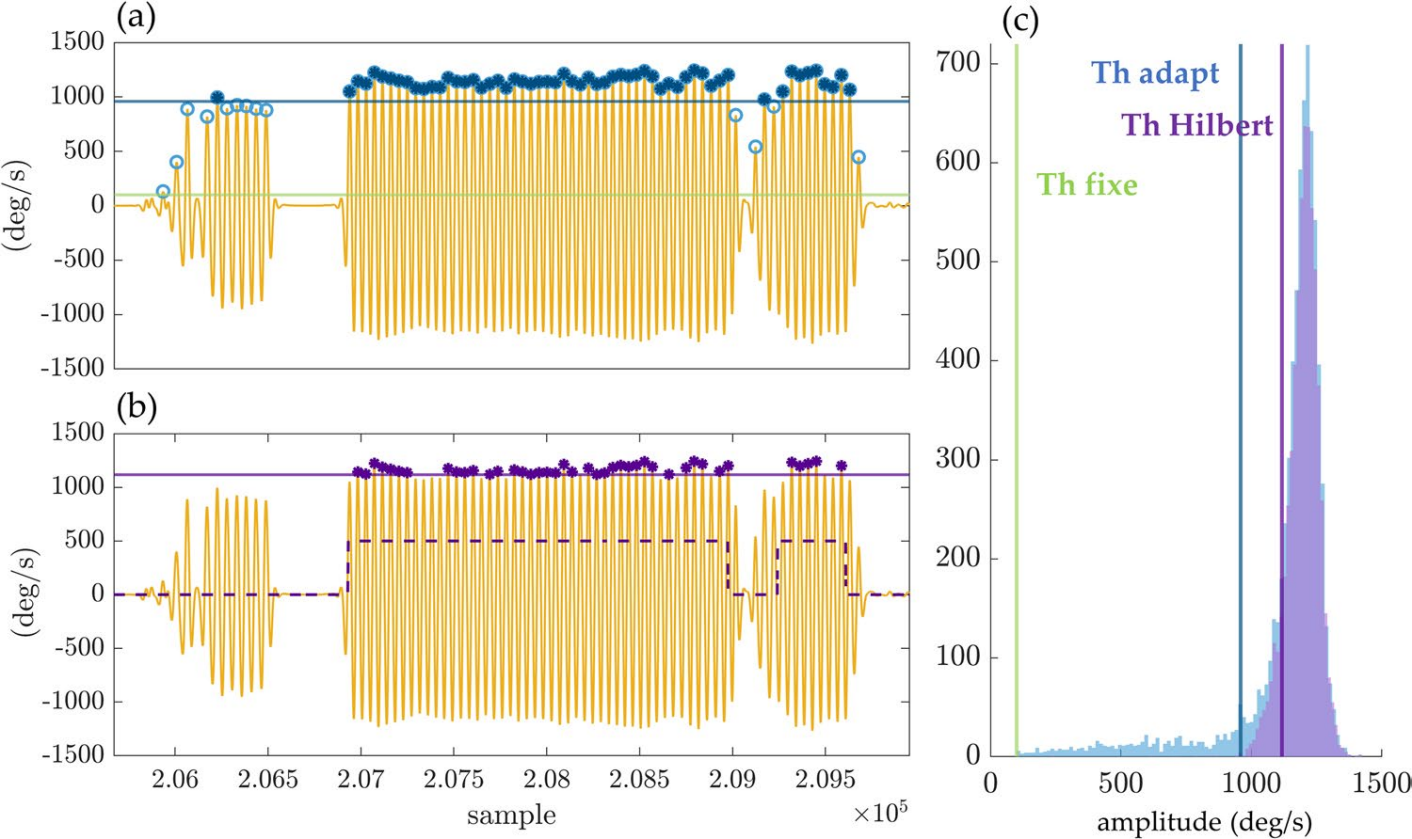
Thresholding methods for peak selection, example based on data from one subject: (**a**) the filtered signal *ωN-LPF-CWT* (orange) is shown, as well as the obtained ${TH}_{adapt}$ (dark blue) and the selected peaks (dark blue starts with amplitudes above the thresholds); (**b**) the Hilbert envelope method is shown with the preselected walking periods (dashed line), and the selected peaks (dark purple dots); (**c**) amplitude distributions of the peaks, and the thresholds obtained for each of the three tested methods (${TH}_{fixed}$ (green), ${TH}_{adapt}$ (blue) and ${TH}_{hilbert}$ (purple)). The histograms of the peaks obtained for the *fixed* and *adaptive* thresholds are identical. In fact, ${TH}_{adapt}$ is defined as the ${10}^{th}$ percentile of the distribution obtained after applying the fixed-threshold ${TH}_{fixed}=100^\circ /s$

From the processed gyroscope angular velocity signal (*ωN-LPF-CWT,* Fig. 2), the time of occurrence $${tp}_{i}$$ of each peak ($${p}_{i})$$ with amplitudes higher than a specific threshold are selected as potential stride-related temporal events. These peaks ($${p}_{i})$$ correspond to mid-swing events when the gyroscope norm is used as input. The heel-strike events, on the other hand, are detected when the acceleration norm is used. Three amplitude thresholding methods were implemented and tested in this study. The first method is based on a fixed threshold $${TH}_{fixed}=100 (^\circ /s)$$, ($${TH}_{fixed}=0.5 \left(g\right),$$ when the acceleration is used as input signal). Both thresholds were chosen in a conservative manner to allow detection of stride-related temporal events for a wide range of walking speeds [38]. The second approach relies on an adaptive threshold ($${TH}_{adapt}$$) based on the percentile of the obtained amplitude distribution of peaks detected above the fixed threshold ($${TH}_{fixed}$$, Fig. 4). Finally, in the third method, we applied the *Hilbert* transform to pre-select potential walking bouts. The *Hilbert* envelope of the filtered signal *ωN-LPF-CWT* is computed for a pre-selected of potential walking periods [39]. Then, the threshold ($${TH}_{hilbert}$$) is defined as the percentile of the amplitude distribution of all peaks in the pre-selected bouts (Fig. 4). Percentile values from 1 to 50%, with increment of 2.5%, were tested through receiver operating characteristic curve (ROC) for the two adaptive thresholding methods.

Figure 4a–b shows the filtered signal *ωN-LPF-CWT* and the derived thresholds ${TH}_{adapt}$ (blue) and ${TH}_{hilbert}$ (purple), as well as the selected peaks (dark dots with amplitude above the thresholds), for data recorded in one subject. Figure 4c shows the amplitude distributions of the peaks and the thresholds obtained for each of the three methods tested (*fixed*, *adaptive* and *Hilbert*). The fixed-threshold ${TH}_{fixed}$ is lower than the other two because it is less restrictive. In contrast, the adaptive threshold ${TH}_{hilbert}$ is higher because of the pre-selected walking periods (dashed line in Fig. 4b).

#### Strides and walking bouts detection using one IMU (one foot)

The next stage consists of detecting the actual strides from peaks ${p}_{i}$ and identifying the beginning/end of the walking periods. Successive peaks with duration $\Delta {t}_{i}= {tp}_{i+1}- {tp}_{i}$ $(i=1,N-1$) lower than the adaptive *duration threshold* (${TH}_{d}$) are considered as part of the same walking period. When one IMU is used, the threshold ${TH}_{d}$ is initialized with a fixed value of 5 s. Then, the threshold is updated at each iteration using the formula ${TH}_{d}=3+average\left(Dstride\right)(s)$, with $Dstride$ defined as the average duration of the previous strides that belong to the current WB. ${TH}_{d}$ is designed to adapt to the cadence of the current walking period, which improves the robustness of the stride detection under real-life conditions. The algorithm is also designed to be resilient to short breaks or occasional undetected stride-related peaks (e.g., during turning, gait asymmetry) by accepting a maximum duration of 5 s between peaks (Fig. 2). The threshold ${TH}_{d}$ is chosen to detect slow walking (minimum cadence around 40 steps/min, corresponding to about a stride duration of 3 s). Finally, only the walking episodes that contained at least two consecutive right and left strides (or five steps) were considered as true locomotion [40].

#### Steps and walking bouts detection using two IMU (both feet) 

First, the algorithm explained in the previous sections is applied to the right and left IMUs' signals independently. Thus, the thresholds for the peak selection obtained for the right and left foot might be slightly different to better adjust to asymmetric gait patterns. As the right and left signals are synchronized, the peaks detected on the right (*ωN-LPF-CWT-right*) and left (*ωN-LPF-CWT-left*) processed angular velocity norms are merged to obtain the right and left mid-swing events. Taken together, these events correspond to the step detection. The maximum duration accepted between peaks within the same walking period is reduced to 3.5 s [10]. Finally, the WBs are detected using the threshold ${TH}_{d}$ adapted for step-related peaks (${TH}_{d}=1.5+average\left(Dsteps\right)(s)$, with $Dsteps$ the average duration of the previous steps that belong to the current WB).

## Validation

The performance of the WB classification was evaluated against the reference (INDIP pressure insoles) by calculating sensitivity, specificity, precision, and accuracy as follow (Eq. 2).

2 $$\begin{array}{c}sensitivity= \frac{TP}{TP+FN}, specificity= \frac{TP}{TP+FP}\\ accuracy= \frac{TP+TN}{Total}, precision= \frac{TN}{TN+FP}\end{array}$$

Each binary vector is sampled at 40 Hz, and a value of 0 or 1 is assigned every second for no locomotion or locomotion, respectively. The true positives (TP), true negatives (TN), false positives (FP), and false negatives (FN) were defined by comparing the binary vector of the reference with the output of the walking detection algorithms (0: no locomotion, 1: locomotion).

## Results

### Validation & ROC curves 

**Fig. 5:**
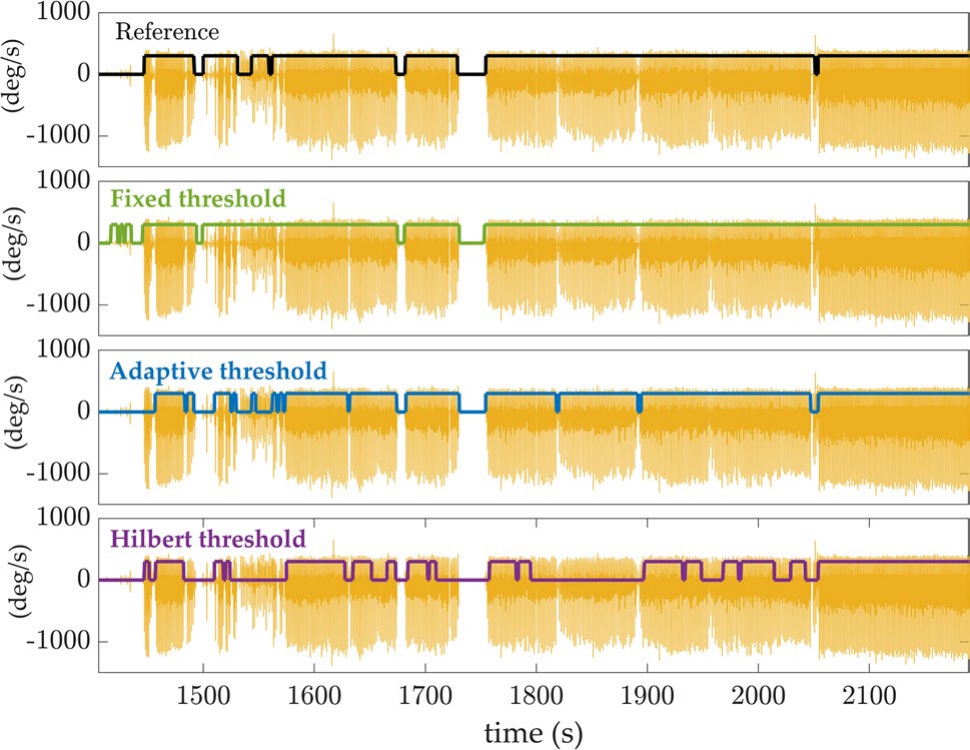
Illustration of walking detection using the different thresholding methods on data recorded in one subject: *fixed* threshold (green), *adaptive* threshold (blue) and *Hilbert* threshold (purple). The reference classification, obtained from the plantar pressure of the INDIP system, is displayed in black at the top of the figure

The mean age of the sample was 30 ± 7.2 years (range 24–46) for HY (5 women, 5 men), and 72.8 ± 3.3 years (range 69–78) for HA (3 women, 5 men). The total number of WBs reported by the INDIP system (i.e., the reference system) was 1156 with an average of (mean ± std) 63.9 ± 33.5 and 71.4 ± 36.7 WBs for HY and HA, respectively. An illustrative example of the walking periods detected by the proposed algorithm, using the three thresholding methods, is shown in Fig. 5. The reference classification obtained using the INDIP system is shown in black (top graph, Fig. 5). We note that the fixed threshold method recognizes all movements as WBs. In contrast, the Hilbert method (${1}^{th}$ percentile) is much more restrictive and misclassifies certain walking periods (false negatives). In this example, the adaptive method (${10}^{th}$ percentile) seems to provide the best walking detection.

**Fig. 6:**
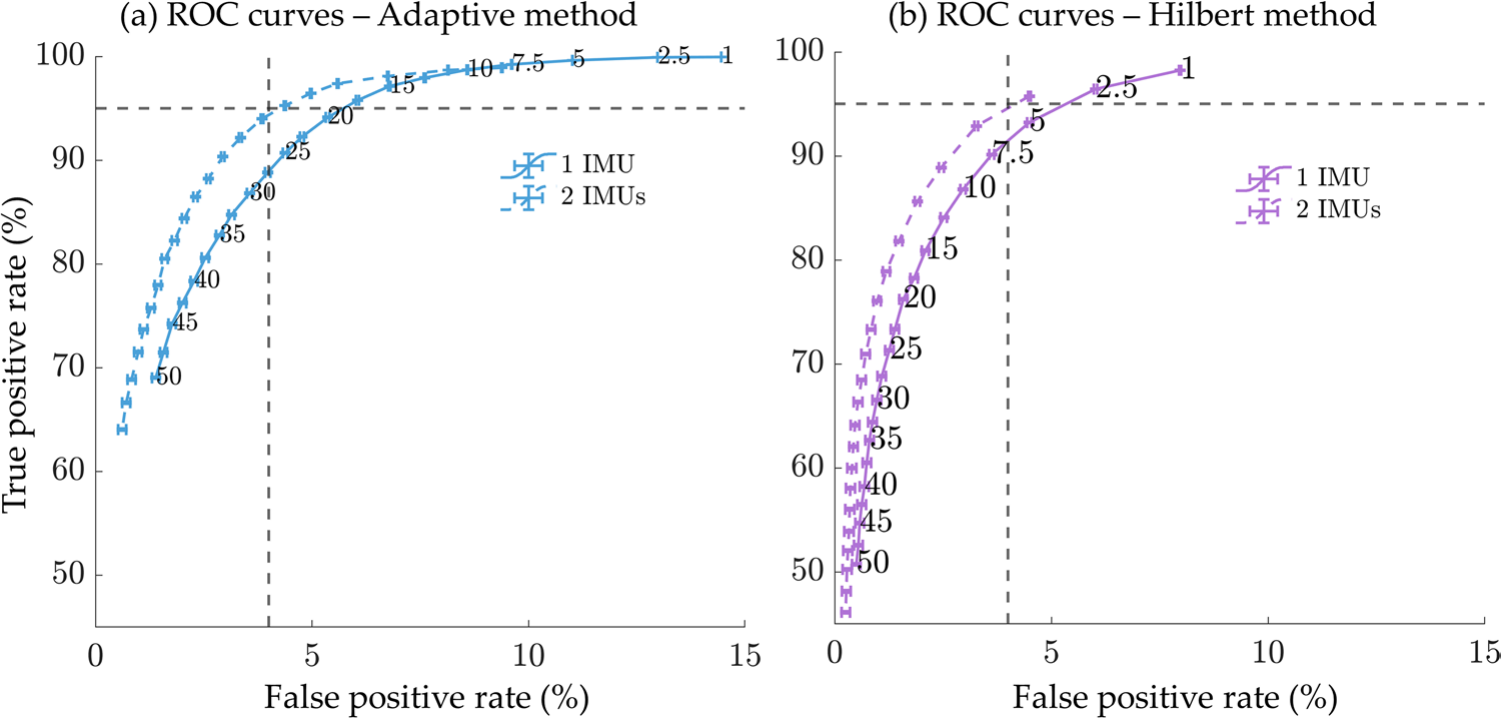
ROC curves for performance evaluation as a function of percentile values from 1 to 50% when the angular velocity norm *ωN* is used as input. The curves are obtained by averaging the results over the 10 subjects when one IMU (continuous line) or two IMUs (dashed line) are used**; (a)** Adaptive threshold method (${TH}_{adapt}$) based on the percentile of the obtained peak amplitude distribution detected above the *fixed* threshold $({TH}_{fixed}=100 (^\circ /s))$*;* (**b**) Hilbert method: The threshold (${TH}_{hilbert}$) is defined as the percentile of the peak amplitude distribution in the pre-selected walking bouts. The horizontal and vertical dashed lines correspond to a 95% true positive rate and a 4% false positive rate, respectively

The adaptive (${TH}_{adapt}$) and Hilbert (${TH}_{hilbert}$) methods depend on the percentile value for threshold selection. To evaluate the influence of this parameter on classification performance, we computed the ROC curves for different percentile values from 1 to 50% with 2.5% increments. Figure 6 shows the average ROC curves calculated for the 18 subjects when one IMU (one foot) or two IMUs (two feet) are used. For the adaptive (${TH}_{adapt}$) method, the best performances are obtained for percentile values between 10% and 12.5%. A higher percentile value would result in a lower sensitivity (some WBs would not be detected). Conversely, a percentile value of less than 10% would result in an increase in the false positive rate (FPR, 1-specificity). As mentioned in the previous section, the Hilbert method (${TH}_{hilbert}$) is more restrictive. The FPR is below 5% for all percentile values tested. However, the sensitivity drops sharply when the percentile value is too high (Fig. 6b). Consequently, the ${TH}_{hilbert}$ should be low (maximum 1%) to avoid a high number of missing walking periods. The algorithms based on two IMUs (right and left foot) demonstrate slightly higher performances (Supplementary material A; Table 1), mainly in terms of specificity. Indeed, the ROC curves obtained with a single IMU are shifted towards a higher false positive rate as seen in Fig. 6. The same procedure was applied to the accelerometer data, and the ROC curves can be found in Supplementary material B.

**Table 1:** The results of the walking detection algorithms based on one IMU (gyr: gyroscope; and acc: accelerometer) for the three different thresholding methods. The ${10}^{th}$ percentile is used for the adaptive method, and the ${1}^{th}$ percentile for the Hilbert approach

		Acc (%) mean (std)	Prec (%) mean (std)	Sen (%) mean (std)	Spe (%) mean (std)
**Gyr**	${TH}_{fixed}$	90.2 (4.6)	69.5 (16.5)	99.9 (0.0)	84.5 (8.3)
	${TH}_{adapt}$	92.8 (3.9)	76.1 (17.1)	98.7 (1.7)	91.4 (5.2)
	${TH}_{hilbert}$	92.9 (3.5)	76.2 (18.5)	98.2 (2.3)	76.2 (18.5)
**Acc**	${TH}_{fixed}$	94.7 (2.1)	81.6 (9.8)	96.4 (4.8)	92.0 (5.8)
	${TH}_{adapt}$	94.9 (2.0)	86.2 (9.5)	91.4 (5.9)	95.9 (2.1)
	${TH}_{hilbert}$	94.4 (2.6)	85.6 (11.2)	91.2 (5.6)	96.1 (2.0)

Table 1 summarizes the performance obtained for one IMU and for the *fixed*, *adaptive,* and *Hilbert* methods. Based on the ROC curves, we used ${10}^{th}$ percentile for the *adaptive* method, and ${1}^{th}$ percentile for the *Hilbert* approach. When using the gyroscope signal as input, the *adaptive* and *Hilbert* methods yield similar results with an accuracy of 93 ± 4%, precision of 76 ± 17%, sensitivity of 98 ± 2%, and specificity of 91.5 ± 4%.

Not surprisingly, the method based on the fixed threshold is very sensitive (almost 100%), which means that all WBs are correctly detected. However, given the lower values for specificity (< 70%) and precision (< 90%), other movements might be misclassified as walking. In general, the results obtained when using the gyroscope signal as input show higher sensitivity and slightly lower precision compared to the acceleration signal (Table 1). Thus, using the gyroscope signal could result in a higher number of false positive WBs, but a lower number of missing walking periods (FNs).

## Discussion

### Walking detection

The walking and stride/step detection algorithms using foot IMU data performed well when applied to a challenging database of 2.5 h of free-living activities. Detection of walking based on a single foot gyroscope signal was achieved with high accuracy (> 90%) and sensitivity (> 98%) for the three threshold methods tested (Table 1). In addition, when adaptive thresholds were used for peak selection (i.e., *adaptive* and *Hilbert* methods), the precision and specificity were over 75% and 91%, respectively. As expected, the results were slightly better for the configuration with two IMUs (one on each foot) because the steps are detected when the peaks from the right and left feet are combined, which is consistent with the reference system (Supplementary material A, Table 1). The algorithm, based on a single IMU, allows the detection of the mid-swing events of one foot (stride-related peaks). Consequently, we increased the maximum resting period time within a bout to 5 s, compared to 3 s when steps are detected, as we do not know if the last or first step of a bout is performed with the unequipped foot. Thus, this threshold of 5 s allows longer resting periods within a bout, and successive walking bouts might be merged together, leading to false positive detection compared to the reference system. Similar accuracy, but slightly lower sensitivity was obtained when the acceleration norm was used as input signal (Table 1).

The proposed algorithm demonstrates an efficient peak enhancement procedure and mid-swing events detection (Figs. 3, 4). As explained in the “*Algorithm implementation”* section, the detection of the mid-swing events depends on a signal amplitude threshold. The *fixed* threshold, chosen to a low value, allows high sensitivity at the expense of lower specificity. The specificity of the walking detection is considerably improved by using the two customized thresholds (*adaptive* and *Hilbert* methods). Using the ${10}^{th}$ percentile of the peak amplitude distribution (i.e., *adaptive* method) allows to remove the peaks which most likely do not correspond to walking (Fig. 4). Regarding the *Hilbert* method, the pre-selected walking periods, based on the *Hilbert* envelope, reduce the possibility of false positive detection. This method is more restrictive than the other two (Figs. 4, 5), and the threshold based on the percentile of the peak amplitude distribution in the pre-selected bouts (i.e., ${TH}_{hilbert}$) should be low to avoid a high number of missing strides. Thus, the *adaptive* and *Hilbert* methods have two main advantages: first, the specificity of the walking detection is improved, and second, those thresholds are based on the data distribution without selecting a specific threshold a priori. Therefore, these methods, which are subject-specific, can be well-adapted to different populations (e.g., slow walkers versus healthy subjects). The choice of one of these threshold methods depends on the application and whether researchers prefer high specificity or high sensitivity in the walking detection. If the main goal is to evaluate the patient’s performance at home based on the distribution of walking speed during the day, we recommend using the *adaptive* or *Hilbert* methods with high specificity. However, if the goal is to focus on the amount of activity performed, it is better to choose a very sensitive method, as each movement can be important. In view of our results, we recommend the use of the *adaptive* method, which has the best trade-off between sensitivity and specificity and a lower computational cost than the *Hilbert* envelope calculation.

Moreover, in this study, we have shown that the gyroscope and accelerometer signals can both be used as input to the walking detection algorithm. Using the gyroscope signals is an interesting option since this allows to subsequently extract gait parameters at each walking bout using dedicated algorithms [28]. However, power consumption of the gyroscope and consequently battery life of the wearable system are critical aspects to consider when conducting long-term monitoring studies. In this context, it might be interesting to collect only accelerometer data, which is less power-consuming than a gyroscope, and characterize the gait by duration of bouts, cadence, and asymmetry if synchronized devices are used on feet.

### Comparison to existing algorithms based on foot-mounted IMUs

In a recent study, Ullrich et al., implemented a novel algorithm for the detection of gait from daily-life recordings [41]. Their approach, based on frequency spectrum analysis, achieved high sensitivity (0.97) on semi-standardized gait tests and high sensitivity (0.98) and specificity (0.96) on laboratory measurements, which is comparable to our results. However, those performance were obtained when the angular rate around the media-lateral axis (considered as the best channel configuration) is used as input signal, requiring therefore a functional calibration. A sensitivity and specificity of 0.89 and 0.81 respectively were reached for the norm of the 3D rate of rotation, which underperforms our results.

Regarding stride detection, a Hidden Markov Model (HMM) approach demonstrated promising results in patients with Parkinson’s disease performing in laboratory walking tests [42]. However, the performance decreased by almost 4% when applied to a free-living recorded dataset. The data-driven HMM approach is highly dependent on the walking bout length, with lower performance for bouts with less than 30 strides [42]. Since short walking bouts (less than 30 s) represent, around 65% of total strides per day [23], this drop in stride recognition performance could be a limitation in a free-living environment. The advantage of the algorithm we proposed in this study is the step- or stride-based detection before the walking detection. Thus, the performance of step/stride detection is independent of the walking bout length.

The main objective of the current study was to validate the walking bout detection method. Therefore, we did not focus on the performance of step/stride detection. However, it is worth noting that unlike the aforementioned approaches, our algorithm enables simultaneous step/stride and walking periods detection. The outcomes of our algorithm provide sufficient information (i.e., the type of activity (locomotion versus non-locomotion), its duration, its intensity, and its frequency) to further analyze the daily physical behavior and their temporal variations [43, 44].

### Strength and limitations

The strengths of this study are evidently associated to the validation of the walking detection algorithm on a challenging database using gyroscope or accelerometer data collected in a real-life like monitoring setting. In addition, the developed algorithm is based on the norm of the gyroscope or accelerometer signal. Therefore, no calibration procedure to correct the sensor orientation is necessary, which makes this algorithm very practical for real life monitoring. However, some limitations must also be acknowledged. Although participants were asked to perform various gait patterns (inside/outside, up/down stairs) to test the algorithm under different conditions, only healthy individuals were included. Therefore, the algorithm needs further validation for very slow walking or gait abnormalities in different clinical populations. One potential limitation is the robustness for very impaired walking patterns, characterized by asymmetry and/or the usage of walking aids. However, we expect our approach to be adaptable to other cohorts as long as the cyclic properties of gait are present in the signals.

Furthermore, we did not evaluate the performance of our algorithm for distinguishing walking to other activities of daily living (e.g. stairs, cycling, running, vacuuming, rope jumping etc.). Future investigations are necessary to verify the robustness of such classification.

### Clinical perspectives

The WB detection methods proposed in this study open new perspectives for gait analysis in real-world conditions which is very relevant for future clinical applications. Once WBs are detected, the spatio-temporal gait parameters such as gait speed, cadence, foot clearance, stride length and variability can be extracted using an IMU (accelerometer and gyroscope) attached to the shoes [25].

The assessment of gait speed under real-life conditions, and the effects of specific interventions or medications on patient mobility are of great interest [8]. Another important clinical application of measuring the spatio-temporal gait parameters under real-world conditions is the evaluation of functional/physical fatigue during the day (i.e., termed ecological fatigue). In the laboratory, fatigue can be quantified using specific walking tests such as the 6-min walk test [45]. However, the measurement of ecological fatigue is still in its infancy and should be explored in future studies. Decreases in activity engagement, walking bout duration and gait speed over the course of the day, as measured by a foot-mounted IMU, may be an interesting direction to investigate further.

## Conclusion

This study validated a robust algorithm for WB detection in a real-life like environment using a foot-mounted IMU. The norms of gyroscope or accelerometer signals are used as input signal to overcome the calibration procedure, which makes this algorithm very practical for real life monitoring. The best results were obtained using the *adaptive* threshold method with sensitivity and specificity of 98% and 91% respectively. For ambulatory monitoring, robust walking detection is required to complete the processing pipeline from raw recorded data to walking/ mobility outcomes. Therefore, this validated algorithm enables robust assessment of locomotion in real-world conditions, opening new perspectives. Ecological fatigue, the effects of medication or rehabilitation periods on physical activity and gait in everyday life can be further explored.

## Supplementary Information

Below is the link to the electronic supplementary material.

Supplementary file1 (DOCX 409 KB)
