# Supplementary material for: A robust walking detection algorithm using a single foot-worn inertial sensor: validation in real-life settings
Source: Med Biol Eng Comput. 2023 Apr 18;61(9):2341–52. doi: 10.1007/s11517-023-02826-x (PMC10412496; doi:10.1007/s11517-023-02826-x)
Supplement: Supplementary file 1 — Supplementary file1 (DOCX 409 KB) [file 11517_2023_2826_MOESM1_ESM.docx]

**Supplementary materials**

A: Walking detection using two IMUs

This appendix shows an illustrative example of the preprocessing steps when two sensors are used (left and right foot) (**Fig. 1**). In addition, the performances obtained when two IMUs are used are provided in the **Table 1** below.

**
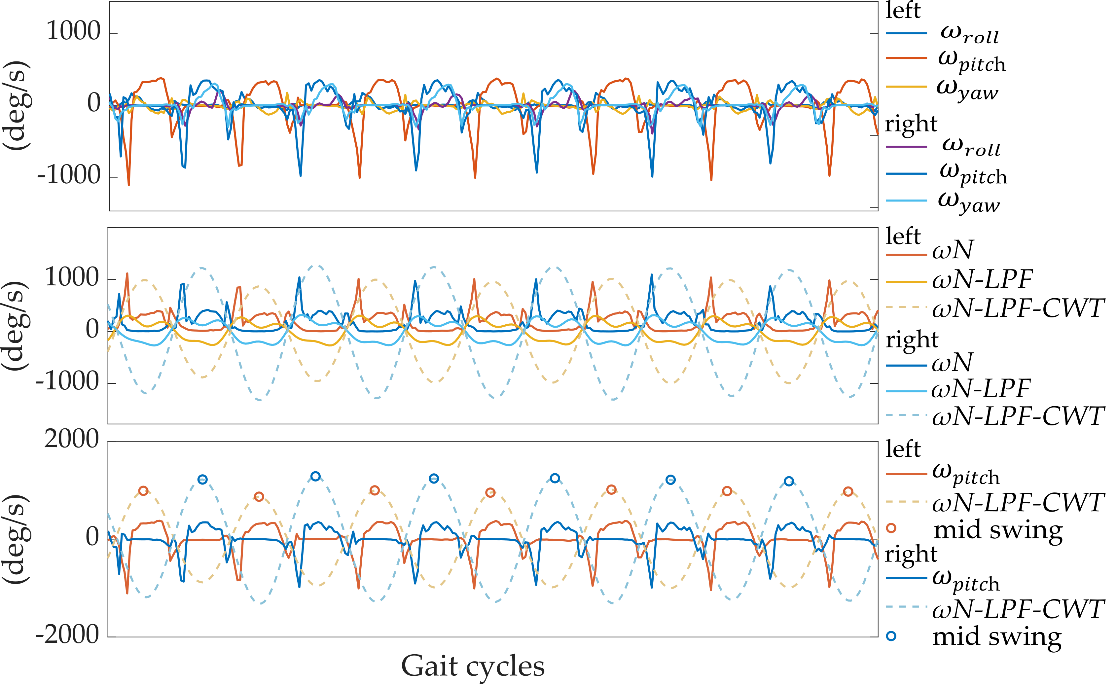
**

**Fig. 1.** Gyroscope signals recorded on two feet during few gait cycles of a subject from the *Mobilise-D* database. The top panel shows the raw angular velocity signals around the three axes. The middle panel shows the raw angular velocity norm (*ωN left and right*, dark red and blue), after detrending and LPF (*ωN-LPF left and right*, light red and blue), and after continuous wavelet transform (*ωN-LPF-CWT left and right*, dashed red and blue lines). The bottom panel shows the pitch angular velocity signals recorded at the two feet. The strides are identified as maxima corresponding to mid-swing events (red and blue circles for left and right respectively).

**Table 1.** The results of the walking detection algorithms based on two IMU (gyr: gyroscope; and acc: accelerometer) for the three different thresholding methods. The ${10}^{th}$ percentile is used for the adaptive method, and the $1^{th}$ percentile for the Hilbert approach.

|  | | **Acc (%)**  mean (std) | **Prec (%)**  mean (std) | **Sen (%)**  mean (std) | **Spe (%)**  mean (std) |
| --- | --- | --- | --- | --- | --- |
| **Gyr** | ${TH}_{fixed}$ | 93.5 (2.7) | 77.3 (12.3) | 99.0 (1.2) | 89.8 (5.9) |
|  | ${TH}_{adapt}$ | 94.9 (2.1) | 83.1 (12.5) | 96.5 (2.2) | 95.0 (2.8) |
|  | ${TH}_{hilbert}$ | 94.9 (2.0) | 83.6 (12.4) | 95.8 (3.2) | 95.5 (2.3) |
| **Acc** | ${TH}_{fixed}$ | 96.0 (1.5) | 87.9 (6.0) | 93.6 (6.8) | 95.0 (4.1) |
|  | ${TH}_{adapt}$ | 95.1 (2.0) | 91.4 (5.4) | 87.0 (7.0) | 97.7 (1.2) |
|  | ${TH}_{hilbert}$ | 94.5 (2.9) | 90.7 (6.8) | 87.0 (6.7) | 97.9 (1.1) |

B: Accelerometer based algorithm

This appendix summarizes the results obtained when the acceleration signal is used as input of the algorithm. To evaluate the influence of the percentile parameter on the classification performance, we computed the ROC curves for different percentile values from 1 to 50%, with 2.5 increments. **Fig. 2** shows the average ROC curves calculated for the 10 subjects in the *Mobilise-D* study. In contrast to the gyroscope-based approach, the ROC curves do not reach the upper left quadrant with a sensitivity of more than 95% and a false-positive rate (FPR, 1-specificity) of less than 4%. With the *adaptive* method (${TH}_{adapt}$), the best results are obtained with percentile values between 10% and 5%. The *Hilbert* method (${TH}_{hilbert}$) is more restrictive, with an FPR below 2% for all percentile values tested. However, the sensitivity is also lower for values below 90%. With the *Hilbert* method (${TH}_{hilbert}$), the best performance is obtained for a percentile of 1%.

**
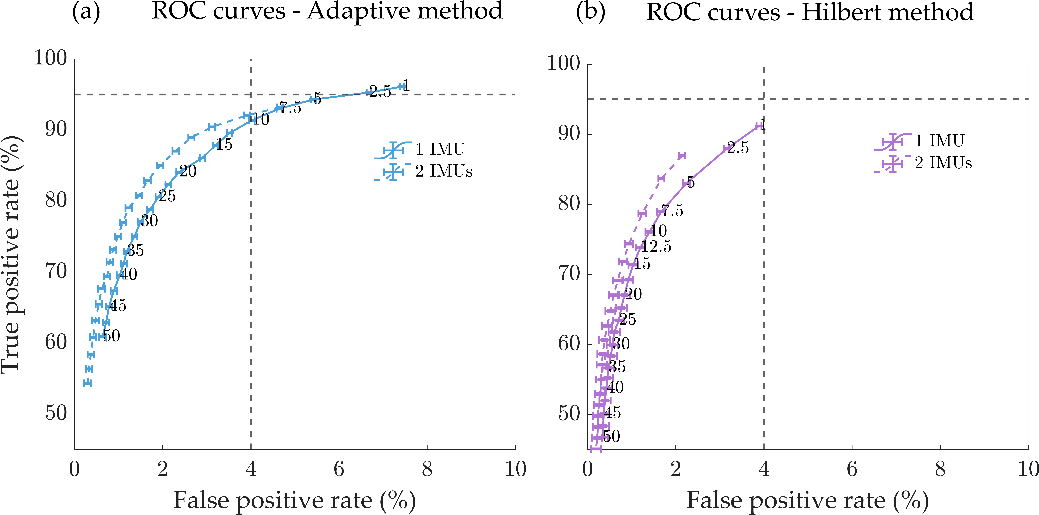
**

**Fig. 2.** ROC curves for performance evaluation as a function of the percentile-values from 1 to 50% when the accelerometer signal is used as input. The curve is obtained by averaging the results across the 10 subjects of the MobilizeD study when one IMU (continuous line) or two IMUs (dashed line) are used; (a) Adaptive thresholding method (${TH}_{adapt}$) based on the percentile of the obtained peak amplitude distribution detected above the fixed-threshold $({TH}_{f}=0.5 g)$; (b) Hilbert method, the threshold (${TH}_{hilbert}$) is defined as the percentile of the amplitude distribution of all the peaks in the pre-selected walking bouts. The horizontal and vertical dashed lines correspond to 95% true positive rate and 4% false positive rate respectively.
